
JOURNAL INFORMATION
==============================
NLM Title Abbreviation: ScientificWorldJournal
Journal ID: ScientificWorldJournal
NLM Journal ID: 101131163
Journal ID: TSWJ

The Scientific World Journal

ISSN: 2356-6140
EISSN: 1537-744X
Publisher: Wiley

ARTICLE INFORMATION
==============================
PMCID: PMC6084193
PMID: 30147506
DOI: 10.1100/tsw.2001.176
Article ID: 542804
Article version: 1
Subjects: Short Report

A Mitochondria-Initiated Apoptotic Pathway

Wang Xiaodong
Howard Hughes Medical Institute, Department of Biochemistry, University of Texas Southwestern Medical School, 5323 Harry Heines Boulevard, Dallas, Texas 75235-9038, USA
Publication date: 2001
Electronic publication date: 2001 Apr 4
Volume: 1
First page: 49
Last page: 49
Copyright: Copyright © 2001 Xiaodong Wang.
Copyright year: 2001
License: This is an open access article distributed under the Creative Commons Attribution License, which permits unrestricted use, distribution, and reproduction in any medium, provided the original work is properly cited.
License URL: https://creativecommons.org/licenses/by/3.0/

==============================
Miami *Nature Biotechnology* Short Reports
*TheScientificWorld* (2001) 1(S3), 49SR
ISSN 1532-2246; DOI 10.1100/tsw.2001.176

### A MITOCHONDRIA-INITIATED APOPTOTIC PATHWAY

Xiaodong Wang

Howard Hughes Medical Institute, Department of Biochemistry, University of Texas Southwestern Medical School, 5323 Harry Heines Boulevard, Dallas, Texas 75235-9038
xwang@biochem.swmed.edu

Emerging evidences indicate that in mammalian cells, mitochondria are not only metabolic centers that provide energy and other essential molecules to sustain life, but also key regulatory centers for cell death. When cells are insulted by apoptotic stimuli, mitochondrial homeostasis is interrupted resulting in releasing of proteins that normally reside in the intermembrane space of mitochondria. The roles of two such proteins in apoptosis have been defined in molecular details. One protein is cytochrome c that normally functions in electron transfer chain in mitochondria. The release of cytochrome c into the cytosol triggers a chain of molecular events mediated by Apaf-1 protein that causes the activation of intracellular protease, caspases. Another novel protein, Smac, is also released from mitochondria to cytosol similar to cytochrome c and functions in apoptosis by removing the inhibitory activities of IAPs, a family of proteins that attenuate apoptosis by inhibiting the activity of caspases.

In this meeting, I will discuss the detailed biochemical mechanism how these proteins interact with each other to determine the life and death of cells.
